# Levels and socioeconomic correlates of nonmarital fertility in Ghana

**DOI:** 10.1371/journal.pone.0247189

**Published:** 2021-02-19

**Authors:** Samuel H. Nyarko, Lloyd Potter

**Affiliations:** 1 Department of Demography, College for Health, Community & Policy, The University of Texas at San Antonio, San Antonio, Texas, United States of America; 2 Department of Population and Behavioral Science, School of Public Health, University of Health and Allied Sciences, Hohoe, Ghana; University of Western Australia, AUSTRALIA

## Abstract

Childbirth outside marriage has several negative implications for the well-being of children, women, and families globally. In sub-Saharan Africa, however, the phenomenon appears to be under-studied. In this study, we examine the levels and socioeconomic correlates of nonmarital fertility in Ghana. Using pooled data from the 2003, 2008, and the 2014 Ghana Demographic and Health Surveys, logistic regression models were used in determining significantly predictive factors of nonmarital fertility. The results show that nonmarital fertility levels have been on the rise over time without any sign of reduction (24.0%, 33.0%, and 40.0% for 2003, 2008, and 2014, respectively). Some socioeconomic characteristics are linked to nonmarital fertility levels with women without formal education, women from poor households, and self-employed women having significantly higher nonmarital fertility risks. Also, older unmarried women, women who have an early sexual debut, cohabiters, women with unmet need for family planning are all associated with considerably higher risks of nonmarital childbearing. A few significant regional disparities also exist, with the Central Region having higher whereas the Upper West Region has lower risks of nonmarital fertility compared to the Greater Accra Region. Childbirth outside marriage is a social concern among women in Ghana. The findings have possible implications for bridging socioeconomic disparities among unmarried women.

## Introduction

Nonmarital fertility–births happening outside marriage–is not considered to be new in both developed and developing countries globally. It is considered as a global family transition process that may not go away [1]. It has been shown that some sub-Saharan African countries such as Rwanda, Ghana, and Liberia among others, have seen a significant increase in nonmarital fertility levels over the past few decades, and in some of these countries, there seems to be no evidence of abating [2]. The high levels of nonmarital fertility among sub-Saharan African countries have been strongly linked to rising age at first marriage and early initiation of sexual activity as well as low use of contraception or abortion services in the sub-region [2, 3].

Understanding the dynamics of nonmarital fertility is necessary because nonmarital childbearing may have several demographic, socioeconomic, and health implications for the child, mother, and the family. For instance, steady increases in nonmarital childbearing levels may lead to a stall in fertility transition, which has been adequately shown among many sub-Saharan African countries [4–6]. As well, evidence shows that being born outside marriage proffers a higher risk of under-five mortality in sub-Saharan Africa [7, 8]. Single mothers may also face some social consequences, such as shame and social stigmatization [9, 10], as well as difficulties in finding a suitable partner [11]. Economically, single mothers may face difficulties in financial or childcare assistance [12]. Despite this, nonmarital fertility or childbearing has remained greatly under-studied in sub-Saharan Africa. In this study, we examine the levels and possible socioeconomic predictors of nonmarital fertility in Ghana. This study ultimately provides an understanding of the levels and trends of childbirths outside marriage and the role of socioeconomic inequalities in Ghana. This study provides evidence of possible implications for reducing nonmarital fertility levels in the country.

## Data and methods

### Data source

This study is based on pooled data from three consecutive waves of the Ghana Demographic and Health Surveys (GDHS) (2003, 2008, and 2014). Conducted as part of the global demographic and health surveys program, the GDHS provides data on the full birth history of women of reproductive age (15–49 years) and for this reason, it provides the best data for this research. In the 2003 wave of the GDHS, 5,691 women of reproductive age and 5,015 men aged 15–59 were interviewed from 6,251 households in 412 clusters across Ghana. The 2008 wave also interviewed 4,916 women and 4,568 men from 6,141 households in 412 clusters nationwide. The 2014 wave comprised 9,396 women and 4,388 men that were selected from 11,835 households covering 427 randomly sampled clusters.

The survey draws on a multistage (two-stage) sample design based on the sampling frame of the 2000 and 2010 decennial Ghana Population and Housing Censuses. A two-stage sampling procedure was used that involves randomly selecting clusters during the first stage and systematically sampling households from the clusters in the second stage [13–15]. The GDHS is an open data source that can be obtained from The DHS Program data repository through free registration and does not require an ethical statement. The unit of analysis is individual women who were not legally married or in formal marital union. These women were either cohabiting or never married and, thus, any childbearing occurring among them was considered as nonmarital childbearing because they were not legally married at the time of childbirth. As such, a total sample size of 8,319 never-married women was attained for analysis, comprising 1,930, 2,135, and 4,254 for 2003, 2008, and 2014, respectively.

### Variables and measurements

The outcome variable of the current study is nonmarital fertility measured as a binary outcome. In this study, terms such as childbearing and childbirth were purposively used interchangeably to refer to fertility. This was generated from the number of children ever born (a count variable) by women who were not legally married or in a formal union. Respondents who had at least one child were considered to have nonmarital fertility (assigned 1), else, they never experienced nonmarital childbirth (assigned 0). The main predictor variables of interest are individual-level socioeconomic characteristics such as educational attainment (no education, primary, secondary/higher), wealth status (poor, middle, rich), work status (working, not working), employer status (self-employed, someone else) and employment period (all year, seasonal). The control predictors used here include demographic characteristics such as current age of the respondents (<20, 20–29, 30–39, 40–49), age at sexual debut (<20, 20+), union status (never married, cohabiting), ethnicity (Akan, Ga/Dangme, Ewe, Mole-Dagbani, Others), religious affiliation (Christianity, Islam, Others), sex of household head (male, female), family planning need (no unmet need, unmet need), survey year (2003, 2008, 2014), place of residence (rural, urban) and region of residence.

### Analytic procedure

Univariate and bivariate analyses were initially conducted, and the results were presented in the form of percentages and a Chi-square test of association for background characteristics of the sample. Also, a multivariate analysis was conducted to examine possible associations between nonmarital fertility chances and socioeconomic factors, among others. Logistic regression models were fitted to estimate the population parameters. The logistic regression model is mathematically specified as:

$${\mathrm{logit}(\mathrm{Nonmarital}/\mathrm{No}\;\mathrm{nonmarital}}_{i})=\beta_{0}+\sum \beta \;x_{i}+u$$

Where *i* refers to the nonmarital fertility status of individual women, and *β*_*0*_ refers to the intercept term while the *βx*_*i*_ term represents the individual-level predictors, whereas *u* refers to the error term.

In this analysis, three nested regression models were fitted whereby Model 1 comprised socioeconomic characteristics of the sample while demographic characteristics were controlled in model 2. Model 3, the full model, also included factors of place and region of residence as controls in the analysis. The estimated model parameters were then used to calculate the final odds ratios and 95 percent confidence intervals. The data analysis was conducted using R statistical software (version 3.5.2) [16], and the analysis was weighted using the complex survey design after de-normalizing the standard weight. The de-normalization was performed by multiplying the standard weight by the ratio of estimated total females aged 15–49 in Ghana in the respective survey years [17] to the number of women aged 15–49 interviewed in the respective surveys.

## Results

### Descriptive results

Table 1 presents the descriptive results of the background characteristics of the sample. About 42 percent of the sample of unmarried women were aged 20 to 29 and the majority (84.3%) were less than 30 years whereas barely 5 percent were aged 40–49. About 72 percent of the sample comprised women who had attained secondary school education or higher while only 9.1 percent had no formal education. More than half (51.1%) of the sample lived in rich households while about 28 percent lived in poor households. The sample is also predominantly comprised of women who had never married (72.2%) and about 28 percent cohabiting women. Christians (87.6%) expectedly were predominant in the sample compared to Muslims, traditionalists, and other miscellaneous religious denominations.

**Table 1:** Background characteristics of the sample.

Characteristics	2003	2008	2014	2003–2014
**Current age**	**%(N = 1,930)**	**%(N = 2,135)**	**%(N = 4,254)**	**%(N = 8,319)**
15–19	50.3(971)	43.6(931)	35.4(1,506)	41.9(3,486)
20–29	38.2(737)	42.6(909)	44.7(1,902)	42.4(3,527)
30–39	8.3(160)	9.7(207)	14.2(604)	11.2(932)
40–49	3.2(62)	4.1(88)	5.7(242)	4.5(374)
**Education level**				
No education	11.3(218)	8.5(181)	8.2(349)	9.1(757)
Primary education	19.8(382)	19.1(408)	17.3(736)	18.5(1,539)
Secondary/higher	68.9(1,330)	72.4(1,546)	74.5(3,169)	72.4(6,023)
**Wealth status**				
Poor	23.0(444)	28.4(606)	30.8(1,310)	28.1(2,338)
Middle	16.0(309)	22.2(474)	22.5(957)	20.8(1,730)
Rich	61.0(1,177)	49.4(1,055)	46.7(1987)	51.1(4,251)
**Union status**				
Never married	77.8(1,502)	71.2(1,520)	69.6(2,961)	72.2(6,006)
Cohabiting	22.2(428)	28.8(615)	30.4(1,293)	27.8(2,313)
**Religious affiliation**				
Christianity	99.2(1,915)	82.5(1,761)	85.1(3,620)	87.6(7,287)
Islam	0.8(15)	11.8(252)	11.4(485)	9.0(749)
Others	0.00(0)	5.7(122)	3.5(149)	3.4(283)
**Ethnicity**				
Akan	59.5(1,148)	58.2(1,242)	55.2(2,348)	57.3(4,767)
Ga-Dangme	8.1(156)	7.3(156)	8.5(361)	8.0(665)
Ewe	13.4(259)	14.0(299)	15.2(647)	14.3(1,190)
Mole-Dagbani	8.4(162)	11.1(237)	11.0(468)	10.4(865)
Other	10.6(205)	9.4(201)	10.1(430)	10.0(832)
**Work status**				
Working	50.9(982)	57.7(1,232)	58.6(2,493)	56.4(4,692)
Not working	49.1(948)	42.3(903)	41.4(1,761)	43.6(3,627)
**Age at sexual debut**				
<20	81.4(1,571)	82.1(1,753)	79.9(3,399)	81.0(6,738)
20+	18.6(359)	17.9(382)	20.1(855)	19.0(1,581)
**Sex of household head**				
Male	51.6(996)	52.6(1,123)	54.0(2,297)	52.9(4,401)
Female	48.4(934)	47.4(1,012)	46.0(1,957)	47.1(3,918)
**Residence**				
Urban	61.6(1,189)	53.1(1,134)	55.5(2,361)	56.2(4,675)
Rural	38.4(741)	46.9(1,001)	44.5(1,893)	43.8(3,644)
**Region**				
Western	10.5(203)	6.6(141)	11.9(506)	9.7(807)
Central	6.6(127)	8.9(190)	9.4(400)	8.5(707)
Greater Accra	21.6(417)	18.9(404)	21.0(893)	20.4(1,697)
Volta	7.6(147)	10.0(213)	8.4(357)	8.8(732)
Eastern	9.7(187)	10.3(220)	10.4(443)	10.2(849)
Ashanti	24.7(477)	25.1(536)	19.4(825)	22.7(1,888)
Brong Ahafo	10.1(195)	9.8(209)	9.1(387)	9.6(799)
Northern	3.8(73)	5.6(120)	6.3(268)	5.4(449)
Upper East	1.7(33)	3.1(66)	2.6(111)	2.6(216)
Upper West	3.7(71)	1.7(36)	1.5(64)	2.1(175)

Source: GDHS 2003–2014

For ethnicity, the Akans (57.3%) represented more than half of the sample while the Ga-Dangmes formed the least (8.0%). Likewise, employed women (56.4%) comprised more than half of the sample compared to unemployed women. The majority of the women in the sample had their sexual debut before age 20 (81.0%). More than half of the sample lived in male-headed households (52.9%) or inhabited urban settings (56.2%). Also, more than one-fifth of the total sample resided in the Greater Accra Region (20.4%) as well as the Ashanti Region (22.7%), while the least resided in the Upper West Region (2.1%).

### Nonmarital fertility levels in the sample

**Fig 1:**
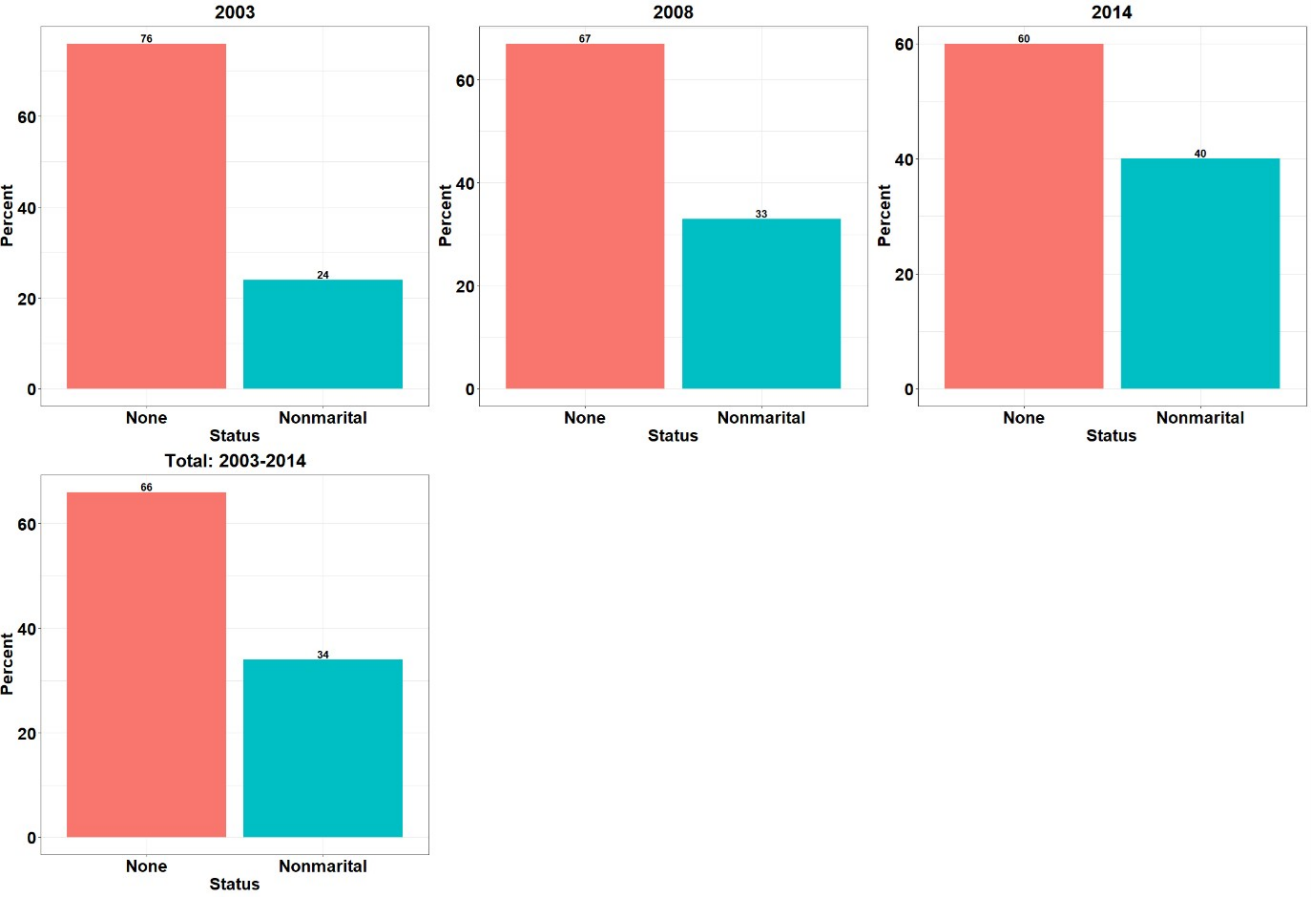
Levels of nonmarital fertility in Ghana: GDHS 2003–2014.

Descriptive results on nonmarital fertility levels are presented by background characteristics of the sample and survey year. The prevalence of nonmarital fertility is presented by survey year for the study period (Fig 1). The nonmarital fertility level among women in the sample was 34.0 percent for the study period. Also, nonmarital fertility levels appeared to soar considerably over time from 24.0 percent in 2003, 33.0 percent in 2008 to about 40.0 percent in 2014.

Furthermore, Table 2 presents a summary of the descriptive results on nonmarital childbearing by background characteristics of women in the sample. Nonmarital childbearing appeared to be most prevalent among older women– 30–39 (80.5%) and 40–49 (94.7%)–but least among teenage women (7.2%). Nonmarital childbearing was also highest among women who had no formal education (59.5%) compared to women with primary and secondary school education or higher.

**Table 2:** Nonmarital fertility levels by background characteristics.

Characteristics	2003%(N = 1,930)	2008%(N = 2,135)	2014%(N = 4,254)	2003–2014%(N = 8,319)
**Current age**	**p = 0.000**	**p = 0.000**	**p = 0.000**	**p = 0.000**
15–19	5.1[3.7, 6.7]	6.8[5.1, 8.7]	9.3[7.1, 12.0]	7.2 [6.1,8.4]
20–29	31.8[27.5, 36.3]	43.1[39.1, 47.0]	43.4[40.0, 46.8]	40.7 [38.5, 42.9]
30–39	77.2[68.6, 84.5]	78.8[72.1, 84.5]	82.6[78.9, 85.9]	80.5 [77.4, 83.3]
40–49	91.7[80.1, 97.8]	95.3[89.1, 98.5]	95.3[91.4, 97.8]	94.7[91.8, 96.8]
**Education level**	**p = 0.000**	**p = 0.000**	**p = 0.000**	**p = 0.000**
No education	39.3[32.2, 46.7]	62.4[54.9, 69.5]	73.5[67.4, 79.0]	59.5 [55.2, 63.6]
Primary education	31.5[26.7, 36.5]	46.7[41.2, 52.2]	50.3[45.9, 54.7]	44.1 [41.1, 47.1]
Secondary/higher	19.4[16.7, 22.2]	25.7[23.2, 28.3]	33.8[30.9, 36.7]	27.6 [25.9, 29.4]
**Wealth status**	**p = 0.000**	**p = 0.000**	**p = 0.000**	**p = 0.000**
Poor	33.3[28.7, 38.1]	42.3[37.6, 46.9]	44.6[40.4, 48.9]	41.5 [38.8, 44.3]
Middle	28.1[22.4, 34.1]	40.6[35.6, 45.7]	50.7[47.0, 54.2]	42.7 [39.9, 45.5]
Rich	19.5[16.3, 22.8]	23.9[20.8, 27.2]	31.6[28.0, 35.3]	25.5 [23.5, 27.5]
**Union status**	**p = 0.000**	**p = 0.000**	**p = 0.000**	**p = 0.000**
Never married	8.1[6.8, 9.6]	12.0[9.8, 14.5]	17.6[15.5, 19.8]	13.2 [11.9, 14.5]
Cohabiting	79.8[74.7, 84.3]	84.3[80.7, 87.5]	90.1[88.6, 92.6]	86.3 [84.4, 88.1]
**Religious affiliation**	p = 0.406	**p = 0.000**	**p = 0.000**	**p = 0.000**
Christianity	23.4[21.1, 25.8]	32.6[29.7, 35.4]	41.4[38.5, 44.1]	33.6 [31.9, 35.3]
Islam	34.1[32.1, 99.9]	24.5[18.6, 30.9]	20.5[16.2, 25.2]	22.6 [19.0, 26.4]
Others	-	53.8[44.3, 63.1]	67.5[58.0, 76.1]	59.6 [52.8, 66.0]
**Ethnicity**	**p = 0.001**	**p = 0.000**	**p = 0.000**	**p = 0.000**
Akan	27.1[23.9, 30.4]	35.4[31.9, 38.8]	42.0[38.8, 45.3]	35.9 [33.8, 37.9]
Ga-Dangme	26.9[20.4, 34.0]	20.7[14.6, 27.8]	44.5[37.4, 51.7]	32.6 [28.1, 37.3]
Ewe	20.3[15.0, 26.5]	40.6[33.9, 47.5]	47.0[41.1, 52.9]	38.7 [34.8, 42.7]
Mole-Dagbani	17.3[11.7, 24.2]	25.7[19.0, 33.2]	21.3[16.7, 26.3]	22.1 [18.6, 25.8]
Others	14.6[9.8, 20.5]	23.5[17.5, 30.1]	33.8[28.0, 39.8]	25.5 [21.8, 29.3]
**Work status**	**p = 0.000**	**p = 0.000**	**p = 0.000**	**p = 0.000**
Working	37.5[33.9, 41.1]	48.8[45.2, 52.3]	51.6[48.6, 54.7]	47.5 [45.4, 49.5]
Not working	10.0[[8.1, 12.3]	11.3[9.2, 13.8]	23.3[20.6, 26.1]	15.6 [14.1, 17.2]
**Age at sexual debut**	**p = 0.001**	**p = 0.000**	**p = 0.000**	**p = 0.000**
<20	42.0[38.1, 45.9]	51.7[48.0, 55.5]	53.9[50.8, 56.9]	50.6 [48.4, 52.7]
20+	28.6[21.6, 36.3]	33.1[26.7, 39.9]	36.4[31.5, 41.5]	33.7 [30.2, 37.2]
**Sex of household head**	**p = 0.002**	p = 0.876	**p = 0.000**	p = 0.190
Male	20.5[17.7, 23.5]	32.6[29.3, 36.1]	43.6[40.7, 46.6]	34.4[32.3, 36.4]
Female	27.8[24.2, 31.5]	33.0[29.5, 36.6]	35.5[32.1, 38.9]	32.7[30.63, 34.7]
**Residence**	**p = 0.000**	**p = 0.000**	**p = 0.000**	**p = 0.000**
Urban	19.2[16.1, 22.5]	26.2[22.9, 29.7]	35.0[31.4, 38.6]	27.8 [25.8, 30.0]
Rural	31.8[28.0, 35.7]	40.3[36.4, 44.1]	46.5[42.4, 49.7]	40.8 [38.5, 43.2]
**Region**	**p = 0.000**	**p = 0.000**	**p = 0.000**	**p = 0.000**
Western	25.2[18.4, 32.9]	18.3[12.5, 25.4]	40.2[34.9, 45.5]	31.1 [27.0, 35.3]
Central	19.0[9.3, 32.4]	40.1[30.2, 50.5]	45.5[35.2, 56.1]	38.5 [31.7, 45.7]
Greater Accra	19.7[15.3, 24.3]	17.4[12.5, 23.0]	35.4[28.7, 42.5]	25.6 [21.9, 29.5]
Volta	15.4[10.9, 20.6]	45.4[38.0, 52.8]	49.4[42.5, 56.2]	40.6 [36.1, 45.2]
Eastern	22.8[15.6, 31.4]	33.3[26.0, 41.2]	48.9[42.4, 55.3]	37.3 [32.9, 42.0]
Ashanti	31.3[25.9, 37.1]	40.6[34.9, 46.3]	35.9[30.2, 41.8]	36.4 [33.3, 39.7]
Brong Ahafo	34.1[26.5, 42.2]	52.2[42.9, 62.1]	48.3[39.4, 57.2]	46.1 [40.8, 51.5]
Northern	14.8[6.8, 26.2]	16.1[10.6, 26.7]	36.5[27.1, 46.5]	26.0 [20.4, 32.1]
Upper East	15.3[9.4, 22.8]	8.0[3.4, 15.3]	13.7[9.8, 18.2]	11.6 [8.6, 15.0]
Upper West	13.3[4.5, 27.7]	12.0[7.8, 17.2]	11.3[7.1, 16.7]	12.4 [8.0, 17.9]

Source: GDHS 2003–2014; confidence intervals are in the brackets.

Nonmarital childbearing appeared to be more prevalent among women from poor (41.5%) and middle status (42.7%) households than among women from rich households (25.5%). Quite expectedly, nonmarital childbearing was greatly prevalent among cohabiting women (86.3%) than never-married women (13.2%). Nonmarital childbearing was most prevalent among women affiliated with other miscellaneous (59.6%) religious denominations than among Christian and Muslim women.

For ethnic differences, nonmarital childbearing was most prevalent among Ewe women (38.7%) but least among Mole-Dagbani women (22.1%). Also, nonmarital childbearing was highly prevalent among employed women (47.5%) compared to their unemployed counterparts (15.6%). Women who had their sexual debut before age 20 (50.6%) had a higher level of nonmarital childbearing than those who had their sexual debut at ages 20 and above (33.7%). For the type of residence, the level of nonmarital childbearing was higher among rural inhabitants (40.8%) than urban inhabitants (27.8%). Nonmarital childbirth also appeared to be more prevalent in the Brong Ahafo (46.1%), Volta (40.6%), Central (38.5%), and Eastern (37.3%) Regions but was less prevalent in the Upper East (11.6%) and West (12.4%) Regions.

### Multivariate analysis results

Table 3 presents the results of the multivariate analysis. In Model 1, the results show that all but one of the socioeconomic characteristics had a significant association with nonmarital fertility. After controlling for demographic and residential characteristics in Models 2 and 3, respectively, educational attainment, household wealth status, and employer status remained significant, whereas the significance of the employment period attenuated. Work status, however, did not show any significant association across all models. Women who had secondary school education or higher had 52 percent lower odds (CI:0.33, 0.69) of having nonmarital fertility, after taking into consideration all the factors, compared to women who had no formal education. In the same vein, women from rich households had 48 percent lower odds (CI:0.35, 0.76) of having nonmarital fertility compared to their counterparts from poor households. The odds of nonmarital childbearing for self-employed women were at least 2.13 times (CI:1.70, 2.68) compared to women employed by someone else. Seasonally employed women, however, had 36 percent lower odds (CI:0.52, 0.78) of nonmarital childbearing compared to women who were employed all year, although this effect attenuated when demographic and residential characteristics were considered.

**Table 3:** Logistic regression analysis of nonmarital fertility levels in Ghana.

Variables	Model 1	Model 2	Model 3
	OR[95%CI]	OR [95%CI]	OR [95%CI]
**Education level** (Ref = No education)			
Primary education	0.78[0.60, 1.02]	0.88[0.57, 1.36]	0.88[0.57, 1.36]
Secondary/higher	0.44[0.35, 0.56]***	0.48[0.33, 0.68]***	0.48[0.33, 0.69]***
**Wealth status** (Ref = Poor)			
Middle	1.16[0.93, 1.45]	0.99[0.72, 1.37]	1.04[0.73, 1.47]
Rich	0.52[0.42, 0.64]***	0.44[0.32, 0.60]***	0.52[0.35, 0.76]***
**Work status** (Ref = working)			
Not working	0.72[0.48, 1.06]	1.06[0.66, 1.70]	1.09[0.69, 1.72]
**Employer status** (Ref = Someone else)			
Self-employed	6.46[5.51, 7.57]***	2.13[1.70, 2.68]***	2.13[1.70, 2.68]***
**Employment period** (Ref = All year)			
Seasonal	0.64[0.52, 0.78]***	0.98[0.72, 1.32]	0.96[0.71, 1.30]
**Current age** (Ref = 15–19)			
20–29		3.21[2.29, 4.48]***	3.24[2.32, 4.52]***
30–39		10.97[7.26, 16.58]***	11.41[7.50, 17.38]***
40–49		18.29[8.58, 38.96]***	19.92[9.33, 42.49]***
**Age at sexual debut** (Ref = 20+)			
<20		3.19[2.48, 4.10]***	3.19[2.48, 4.12]***
**Union status** (Ref = Never married)			
Cohabiting		8.58[6.47, 11.54]***	8.47[6.29, 11.41]***
**Religious affiliation** (Ref = Christianity)			
Islam		1.23[0.76, 1.98]	1.24[0.76, 2.01]
Others		1.69[0.91, 3.12]	1.71[0.92, 3.16]
**Ethnicity** (Ref = Akan)			
Ga-Dangme		0.86[0.60, 1.24]	1.02[0.67, 1.55]
Ewe		0.70[0.52, 0.94]*	0.84[0.56, 1.24]
Mole-Dagbani		0.90[0.60, 1.37]	1.09[0.69, 1.73]
Other		0.74[0.45, 1.19]	0.82[0.51, 1.34]
**Sex of household head** (Ref = Male)			
Female		1.24[0.98, 1.57]	1.25[0.98, 1.59]
**Family planning need** (Ref = No unmet need)			
Unmet need		1.31[1.01, 1.72]*	1.26[0.96, 1.67]
**Year (Ref = 2003)**			
2008		1.36[0.98, 1.89]	1.35[0.97, 1.87]
2014		1.82[1.35, 2.46]***	1.82[1.35, 2.46]***
**Place of residence** (Ref = Urban)			
Rural			1.16[0.82, 1.61]
**Region** (Ref = Greater Accra)			
Western			1.38[0.85, 2.23]
Central			1.54[1.01, 2.96]*
Volta			1.06[0.66, 1.70]
Eastern			1.36[0.89, 2.06]
Ashanti			1.38[0.93, 2.06]
Brong Ahafo			1.35[0.80, 2.28]
Northern			1.33[0.69, 2.55]
Upper East			0.82[0.39, 1.72]
Upper West			0.54[0.16, 0.90]*
Log-LRT (P-value)	3432.02[p<000]	1771.30[p<0.000]	8.74[p<0.529]

Ref = Reference category; OR = Odds Ratios; Significance: 0.000 “***”; * “0.05”.

Source: GDHS 2003–2014.

In Models 2 and 3, some demographic and residential characteristics as well had a significant association with nonmarital childbearing. Women’s current age had a significant positive association with nonmarital childbearing. The odds of nonmarital childbearing were 3.24 times (CI:2.32, 4.52) for women aged 20–29, 11.41 times (CI:7.50, 17.38) for women aged 30–39, and 19.92 times (CI:9.33, 42.49) for women aged 40–49, compared to women below age 20. The age at sexual debut was also important for nonmarital childbearing, as women who had their sexual debut in their teens (OR:3.19; CI: 2.48, 4.12) had disproportionately higher odds of having nonmarital childbirth compared to those who had their sexual debut at ages 20 and above.

Regarding union status, the odds of nonmarital childbirth for cohabiting women were 8.47 times (CI:6.29, 11.41) compared to never-married women. Religious affiliation, ethnicity, and sex of household head, however, had no association with nonmarital childbirth. The need for contraception was associated with nonmarital childbearing, with women who had an unmet need for family planning having 31 percent higher odds (CI:1.01, 1.72) of nonmarital childbearing compared to women with a met need for family planning. The results also show evidence of a significant increase in nonmarital childbearing over time. The odds of nonmarital childbirth were 34 percent higher in 2008 (albeit insignificant) and 82 percent higher (CI:1.35, 2.46) in 2014 compared to 2003.

Moreover, in Model 3, place of residence, however, appeared to have no significant association with nonmarital childbearing albeit rural women had higher odds compared to urban women. Region of residence showed a significant association with nonmarital childbirth for a few regions. Women residing in the Central Region had 54 percent higher odds (CI:1.01, 2.96), whereas women residing in the Upper West Region had 46 percent lower odds (CI:0.16, 0.90) of nonmarital childbirth compared to women living in the Greater Accra Region.

## Discussion

This study provides enlightening evidence on the levels and socioeconomic underpinnings of nonmarital fertility in Ghana, among other factors. The descriptive findings show that the nonmarital childbearing levels are considerably high and have been steadily on the rise over the study period. This directly supports the argument of Clark et al. [2] that nonmarital fertility levels have been on the rise in some sub-Saharan African countries over the last few decades without any sign of improvement; and with the causes being linked to increasing age at first marriage, early sexual debut and low use of family planning services, among others.

Furthermore, some socioeconomic characteristics appear to play an important role in the levels of nonmarital childbearing in the country. Women’s education attainment is associated with nonmarital childbirth with women having secondary school education or higher being significantly less likely to have a nonmarital child compared to women without formal education. Women who have no formal education appear to have substantially higher risks of nonmarital childbearing. Findings of previous studies regarding the empirical association between educational attainment and nonmarital childbearing in sub-Saharan Africa are mainly conflicting. It is, however, noteworthy that these mixed findings may be due to differential national contextual factors in these countries. This study corroborates a few studies that show a negative association between education and nonmarital fertility [18, 19]. In the Ghanaian context, the reason may be that highly educated women most likely use modern contraception to protect themselves [20], while women who have no formal education have higher risks of nonmarital childbearing probably because they may have higher rates of unplanned pregnancies, compared to their educated counterparts [21]. Conversely, many of the extant studies show a positive association between educational attainment and nonmarital fertility [3, 22–25].

The findings also link nonmarital childbearing chances to household wealth status, whereby women from rich households appear considerably less likely to have a child outside marriage than women from poor households. Women from poor households show a greater risk of nonmarital childbirth and this supports the findings of many related studies in sub-Saharan Africa [3, 18, 19, 25, 26]. Women from rich households have a significantly lower risk of unmet need for family planning compared to women from poor households [27], clearly because they may afford and access sexual and reproductive health services such as family planning and abortion services, information, and education to prevent childbirths outside marriage. On the contrary, there appears to be some evidence associating women from rich households with a significantly higher likelihood of nonmarital childbearing [23].

Additionally, among employed women, the findings show a link between nonmarital childbearing and employer status of unmarried women with self-employed women having higher nonmarital childbearing risks compared to women employed by someone else. It is unclear why self-employed women may face significantly higher risks of nonmarital childbirth; however, further studies may provide a better understanding of this subject. Also, regular working women appear to be associated with higher nonmarital childbirth risks as against seasonal workers even though the significance wanes when demographic and residential characteristics are considered.

Moreover, this study brings to light the important role played by some demographic characteristics of women. The findings show a positive link between women’s current age and nonmarital fertility risks. Older women have a disproportionately higher likelihood of nonmarital childbearing within the sample. Some similar studies in sub-Saharan Africa such as Namibia [18], Nigeria [19], Burkina Faso [25], and South Africa [28] also provide evidence on the positive association between women’s age and nonmarital childbearing risks. This is likely because, in sub-Saharan Africa, particularly Ghana, many older or aging women who are still unmarried may likely give up on marriage and focus on having children outside marriage if they feel they can successfully cater for them. This may be done to avoid being childless by the end of their reproductive period and, in turn, save themselves from the strong social pressures or stigmatization associated with having no child. The age at the sexual debut of women also appears to be important in determining their nonmarital childbearing chances. Sexual debut during the teen years is associated with significantly higher chances of nonmarital childbearing among Ghanaian women. Thus, the timing of sexual debut may have crucial implications for addressing childbirth outside marriage and informing social policies seeking to address this phenomenon.

Union status is also found to be significantly associated with nonmarital childbearing risks among women in the sample. The findings show that cohabiting women may have excessively higher risks of nonmarital childbearing compared to never-married women. The significant association between women’s cohabitation and higher nonmarital childbearing risks has also been shown in Namibia [18]. It is apparent that most of the childbirths outside of recognized marriage in Ghana happen within cohabitation unions and this may reflect the fact that some Ghanaian couples may be using cohabitation as an option for reproduction and companionship if they could not afford the cost of marriage.

As well, an unmet need for family planning is significantly correlated with nonmarital childbearing chances among Ghanaian women. In essence, women with an unmet need for family planning have considerably higher chances of having a child outside marriage compared to women without an unmet need for family planning. Analogously, Garenne and Zwang [3] provide evidence on the association between contraceptive use and reduced nonmarital childbearing risks. Besides, adequate evidence shows that contraceptive use may also reduce total fertility levels considerably [29–31], but not just nonmarital childbearing levels. This is a clear indication that universal access to family planning services may be a key agent in reducing nonmarital fertility levels in Ghana.

In addition, there is evidence of time association with nonmarital childbearing chances in the country. The findings show a steady and considerable increase in nonmarital childbirth risks over the study period. The steady increase in nonmarital childbearing levels over time may have crucial implications for the social wellbeing of many children in the country. Lastly, the study finds a few considerable inequalities in nonmarital childbearing risks in the country. In comparison with their counterparts in the Greater Accra Region, unmarried women residing in the Central Region appear to have considerably higher nonmarital childbearing risks whereas those in the Upper West Region appear advantaged in this regard. At this juncture, it is unclear what the intervening factors maybe, but it is noteworthy that exploring regional contextual factors may be an invaluable starting point.

One potential limitation of this study is that the study is focused on women who had never married. Owing to data inadequacies, however, the study excluded formerly married women and currently married women (ever-married sample) at the time of the survey. Hence, it excludes possible nonmarital childbirths occurring after the dissolution of marriage and among unmarried women who were married at the time of the survey. Notwithstanding, the study provides an important contribution to the inadequate extant literature on nonmarital fertility.

## Conclusions

Nonmarital fertility is becoming a serious social concern among women in Ghana. It appears to steadily increase over time without any evidence of abating. Some socio-economic factors such as educational attainment, household wealth status, and employer status are linked to nonmarital fertility levels in the country, with women without formal education, women from poor households, and self-employed women having disproportionately higher risk levels. Considerably higher nonmarital fertility risks are also evident among older women, women who have an earlier sexual debut, and cohabiting women, indicating the crucial role that may be played by some demographic factors. Unmet need for family planning also may play a significant part in nonmarital fertility levels, coupled with some notable regional disparities in risk levels in the country. The findings may have many possible implications for addressing nonmarital fertility levels including alleviation of socioeconomic inequalities among women, particularly in educational attainment and wealth creation. The findings further imply that women in high-risk groups including unmarried self-employed and older women should be prioritized in policies meant to address nonmarital childbearing. As well, universal access to family planning services and deferment of sexual debut among unmarried women may help to considerably reduce nonmarital childbearing risks in Ghana.
